# Garlic (*Allium Sativum*) Supplementation Improves Respiratory Health but Has Increased Risk of Lower Hematologic Values in Horses

**DOI:** 10.3390/ani9010013

**Published:** 2019-01-02

**Authors:** Markku Saastamoinen, Susanna Särkijärvi, Seppo Hyyppä

**Affiliations:** 1 Natural Resources Institute Finland (Luke), Production Systems, Tietotie 2, 31600 Jokioinen, Finland; susanna.sarkijarvi@luke.fi; 2 Ypäjä Equine College, Opistontie 9, 32100 Ypäjä, Finland; seppo.hyyppa@hevosopisto.fi

**Keywords:** horse, nutrition, respiratory health, dietary supplementation, hematology values

## Abstract

**Simple Summary:**

The hypotheses of this study were that garlic supplementation may help to clear mucus in the airways, but also causes declining hematologic values in prolonged feeding. The results show that long-term supplementation of dried garlic on the level of 32 mg/kg BW improved respiratory health in terms of reduced amount of tracheal symptoms and accumulation of tracheal exudates. However, the garlic supplemented horses showed slightly declining hemoglobin (Hb), hematocrit (HcT) and red blood cells (RBC) values.

**Abstract:**

Garlic (*Allium sativum*) is claimed to have numerous beneficial properties to the health of humans and animals. It is commonly used for example to treat respiratory diseases and infections in horses’ lungs. However, in addition to its possible positive influences, garlic may also have adverse health effects. The hypotheses of this study were that garlic supplementation may help to clear mucus in the airways, but also causes declining hematologic values in prolonged feeding. To our knowledge, this is the first organized study in controlled conditions to show the health effects of garlic supplementation for horses so far. The results show that long-term supplementation of dried garlic on the level of 32 mg/kg BW seemed to reduce the amount of tracheal symptoms and accumulation of tracheal exudates. Additionally, the number of neutrophil cells in the tracheal mucus was numerically smaller in the garlic supplemented horses. However, the garlic supplemented horses showed slightly declining Hb, HcT and RBC values during an 83-day study period. Consequently, it is possible that even low garlic supplementation levels can be detrimental to the horse’s hematology when the supplementation period is long.

## 1. Introduction

Garlic (*Allium sativum*) has been used in the diets of humans and animals for centuries because of its believed positive health effects, which contain many active components [1]. In horse nutrition and care, garlic is typically used to treat respiratory diseases and infections in their lungs, and to provide relief from the symptoms of coughs. In horse stables, the hygienic quality of the air may be poor and airborne dust concentrations may reach levels that are detrimental to horses’ respiratory health [2,3]. The main sources of respirable particles and allergens causing respiratory symptoms are forages and bedding materials [4,5]. The highest dust measurements are observed in winter when the stable doors are closed [6].

The onion family (*Allium* species) is rich in an active component of organosulfur compounds [7] that are associated with the above-mentioned beneficial properties, but, on the other hand, are also reviewed to be associated with toxicosis in mammals [8]. One of those toxins is N-propyl-disulfide, that alters the enzyme glucose-6-phosphate dehydrogenase in red blood cells. This interferes with the cells’ ability to prevent oxidative damage to hemoglobin [9]. Ingestion of onions can cause hemolytic anemia in horses [10]. The toxic effects and the mechanism are reviewed by Hutchison [11] and Pearson et al. [12]. There are scientific papers presenting and reviewing studies on adverse effects of garlic in humans and various animal species, i.e. horses, cattle, birds, rats and dogs [8,11,13,14,15].

Garlic is claimed to have also many other positive effects and is, consequently, often included as a supplemental feed to the diets of horses [7,16]. The benefits are, for example, antidiabetic and antimicrobial as well as antiparasitic properties [17,18,19]. Garlic is also commonly used as an insect repellent [13]. Studies on influences of garlic in horse nutrition are, however, scarce, and the dosage for beneficial effects is not known. Furthermore, there is little information on possible adverse health effects to determine the safe use of garlic for horses. Bergero and Valle [20] concluded that the traditional use of herbs is not always properly based on dosages, and moreover, safety is not automatically provided. Supplements considered safe in humans and other species are not always safe in horses. The authors of a recent study [15] suggested that the usage of garlic as a feed additive should be monitored carefully because of the detrimental effects of overdosing.

There may be a risk of anemia when certain sizes of garlic doses are fed to horses for a long period of time. Pearson et al. [12] reported that a daily dose of dried garlic over 200 mg/kg BW developed indications of Heinz body anemia, and concluded that the potential for garlic toxicosis is present when horses are chronically fed with garlic. However, they had only two supplemented horses in their study. Based on this limited data, the report of The National Academy of Sciences [21] in the U.S. gives presumed and historical safe intakes of 90 and 15 milligrams per kilogram of body weight, and concluded that the threshold level above which the risk of an adverse event will increase significantly is likely to be between 15 and 200 mg/kg BW of dried garlic, potentially depending on the health and oxidative status of the individual horse involved.

It is possible that even low supplementation levels may be detrimental when the period of supplementation is long in duration. Elghandour et al. [15] suggested that herbal supplements not tested in horses have to be evaluated to verify the possible negative side effects, followed by standardization of the dosage. Consequently, data is needed to provide more precise dosing information to obtain the beneficial effects and, on the other hand, to avoid the detrimental effects of garlic. 

The aim of this study was to evaluate the possible positive influence on airway health, as well as possible adverse health effects of garlic supplementation in horses. Our hypotheses were that garlic supplementation may help to clear mucus in the airways, but may also result in decreased hematologic values in prolonged feeding. To our knowledge, there are no studies with this or a larger number of horses carried out in controlled circumstances so far.

## 2. Materials and Methods

### 2.1. Geographical Area and Climate Conditions

The experiment was conducted in the facilities of MTT Agrifood Research Finland (currently Natural Resources Institute Luke) in the south western part of Finland (latitude 60°) during early and mid-winter (November to January) climatic conditions. The average outdoor temperatures were −1.0 °C (−6–3 °C), −6.0 °C (−21–1 °C) and −4.6 °C (−24–1 °C) in November, December and January, respectively. When compared to the long-term weather statistics of the Finnish Meteorological Institute (Climate guide.fi), the temperatures were within the long-term monthly averages.

### 2.2. Horses and Housing Conditions

Twelve Finnhorse mares (aged 5 to 17 years, weighing 595 to 710 kg) were housed in a box stable with an automatic ventilation system in individual stalls (3 m × 3 m) and peat as bedding. The peat was manufactured for use as bedding in horse stalls (Vapo Ltd., Jyväskylä, Finland), and was chosen to be used as bedding because of its beneficial effects on stable air quality [22]. The horses had been stabled in the same stable since they were taken indoors from pastures at the beginning of September. The stalls were manually cleaned daily between 8 and 12 a.m. when the horses were in outdoor paddocks. All feces and wet material were removed and new bedding material was added. 

The experimental design was a randomized block design with repeated measurements. After the first endoscopy, the horses were formed into pairs based on matched health status and upper respiratory tract characteristics as determined by the endoscopy (symptomatic similarity), as reported in our earlier study [22]. The two horses of each pair were then allotted to an experimental group and a control group and placed in the stable so that the stable air conditions were as equal as possible for all pairs of horses (for both groups). The horses had free daily exercise in paddocks in groups (grouped by experimental groups) for four hours, and for one hour of riding or driving. The stable temperatures and humidity, as well as outdoor temperatures and weather conditions were followed and recorded daily at 8 a.m. 

### 2.3. Experimental Feeding

The horses were individually fed three times per day (morning, noon, and evening) with a hay (871 g DM/kg) and oat (883 g DM/kg) diet supplemented with a linseed-molasses-beet pulp mixture (905 g DM/kg, Neomed Ltd, Somero, Finland) at maintenance energy level [23], with a forage-to-concentrate ratio of 80:20. The hay was produced by MTT and was artificially cured (barn drying) to ensure good hygienic and nutritional quality. Based on the chemical analysis, it was of medium nutritional quality [24], and it fulfilled the criteria of good quality dried hay (sensory evaluation of dust, smell, color carried out by experienced researchers). The oats and concentrate mixture were moistened with water to minimize the release of airborne particles from the feeds. In addition, the experimental group (one horse of each matched pair) was supplemented with 20 g of dried garlic flakes (moistened before feeding), corresponding to 0.2 % of the DM intake and 32 mg/kg BW, which is within the safety limits given by The National Academy of Sciences [21]. The supplementation continued for a total of 83 days. The diets were also balanced with a commercial mineral-vitamin mixture (Suomen Rehu Ltd., Seinäjoki, Finland). The palatability of the feeds fed to horses was good, and the horses ate all the hay, concentrates (oats, mixed feed) and the garlic supplementation offered to them, with good appetite. Thus, no feed residuals existed. 

### 2.4. Respiratory Tract Examination and Blood Sampling

Upper respiratory tract (ethmoidal region, pharyngeal openings of guttural pouches, soft palate, larynx and trachea) examination by endoscopy was performed three times during the study (days 0, 41 and 83), and the findings were recorded (found = 1; not found = 0). Tracheobronchial aspirates were drawn at the time of the endoscopy and cytological and a bacteriological (neutrophil cells) evaluation was conducted from the tracheal mucus. The classification of the neutrophil cells in bronchoalveoral smear samples was as follows: none or some single cells (–); single cells and few small pools of cells (+); several large pools of cells (++); abundant pools of cells (+++); and an extreme abundance of cells (++++). 

Blood samples from the jugular vein, as measures of health and wellbeing of the horses, were collected at the same interval as the endoscopic examinations were done. The blood analysis consisted of white blood cells (WBC), red blood cells (RBC), mean cell corpuscular volume (MCV), hematocrit (HcT) and hemoglobin (Hb) contents. The white blood cells were differentiated. All samples were analyzed in the MTT clinical laboratory. The endoscopy examination was done and all samples from the horses were collected by a veterinarian researcher.

In animal handling and sample collection, the European Union directives (1999/575/EU; 2007/526/EU) and national animal welfare and ethical legislation based on the directives above and set by the Ministry of Agriculture and Forestry of Finland were followed carefully. The experimental procedures were evaluated and approved by The Animal Care Committee of MTT Permit 43/2000 before the study was commenced.

### 2.5. Statistical Analysis

The information of the first endoscopy (day 0) was excluded from the data because it was included in the animal pair-variable in the model. The data (samples from horses) were analyzed with a linear model applying the MIXED procedure of the SAS system (SAS Institute Inc., Cary, NC, USA) with the following statistical model: *Y_ijk_ = µ + p_i_ + b_j_ + (p × b)_ij_ + t_k_ + (p × t)_jk_ + (b × t)_jk_ + e_ijk_*, where *Y_ijk_* is the observation, *µ* is the overall mean, *p_i_* is the random effect of *i*th animal pair (*i* = 1…6), *b_j_* is the fixed effect of *j*th feeding (*j* = 1…2), *t_k_* is the fixed effect of the time period (*k* = 2 or 3), and *e_ijk_* is the normally distributed error with a mean of 0 and variance of δ^2^. The terms (*p* × *b*)*_ij_*, (*p* × *t*)*_jk_* and (*b* × *t*)*_jk_* are compound factor effects. The differences were tested with a Tukey’s test. Categorical variables (neutrophil cells in tracheal mucus) and 0/1-variables were not tested statistically, but were presented descriptively, because of the small number of observations and their subjective scoring making them less informative. 

## 3. Results and Discussion

### 3.1. Housing Conditions

The average temperatures and humidity of the stable air in November, December and January are presented in Table 1. These temperatures were mainly within the target indoor temperature range (8–12 °C) in Finland, corresponding also to those reported for the winter months in the same stables in a previous study dealing with stable air quality [22]. However, on three days in December and one day in January, rather low stable temperatures (3–4 °C) were observed. On those days the outdoor temperatures were at their lowest (−20 °C or below). The stable air humidity was at the lowest levels naturally during the days when the outdoor temperatures were the lowest.

### 3.2. Respiratory Health

Data on the neutrophil cells in the tracheal mucus (in terms of categorical variables), and the endoscopy examination findings (0/1-variables) were not tested statistically because of the small number of observations and their subjective scoring, thus being less informative. The garlic supplementation seemed to reduce the tracheal exudate score based on the endoscopy examination (Table 2). The clinical signs disappeared in three of the six horses that were given garlic supplements, while one horse remained without any signs during the study period. Clinical signs remained in two horses from the garlic supplemented group. Concerning the control horses, they remained in three horses, fluctuated in two horses and disappeared in one horse. The present results are supported by Pearson [5], who reported a significant decrease in the respiratory rate in horses supplemented with garlic. 

The tracheobronchial aspirates obtained during the endoscopy contained either scarce or moderate numbers of neutrophils (Table 3). The number of neutrophils in the tracheal mucus was smaller among the group of garlic supplemented horses on day 83. During the study course (days 41 and 83), a large number of neutrophil cells (+++) was found only in two samples in the supplemented group, but in four samples of the six horses in the control group. The neutrophils in the tracheal mucus of the control horses remained high or increased in three individuals during the study, but decreased in two of the supplemented horses (from +++ to +), and in one of them the number increased (from ++ to +++). An elevated number of neutrophils or the detection of Curschaman’s spirals is suggested to correlate with COPD symptoms [25]. However, no horses were diagnosed with COPD in the present study. Nor were any clinical signs of declined health status observed. 

The differences between the groups were small, and because of the small data set and subjective evaluation of the endoscopy findings and incidence of the neutrophil cells, drawing reliable conclusions is not possible. In addition, we used bedding material (peat) low in respirable particles and hay with high hygienic quality. In practical conditions, there are several factors affecting the respiratory health of horses [26]. It is most important to use alternative bedding materials and feeds of high hygienic quality which are free from dust (molds, respirable particles) to reduce airborne dust and aeroallergens in stables [5,22].

At the beginning of the trial, the horses had spent about two months (September–October) in the stable with outdoor exercise sessions as described earlier. The first endoscopic examination at the beginning of the experiment revealed that 9 out of the 12 horses had signs of respiratory symptoms. Thus, moving the horses from the pasture into indoor housing at the beginning of September appeared to expose the horses to respiratory disease because of the air quality in the stable. This is supported by Elfman et al. [6] who found (in Swedish weather conditions comparable to those in Finland) that dust and airborne bacteria levels increase in September compared to other seasons in their study. Also, our previous study [22] reported that respiratory symptoms increased during the study period (October – December) in quite similar environmental circumstances, but that peat as a bedding material might have positive effects on respiratory health because of its beneficial influence on the air quality of the stable.

### 3.3. Blood Analysis

**Figure 1:**
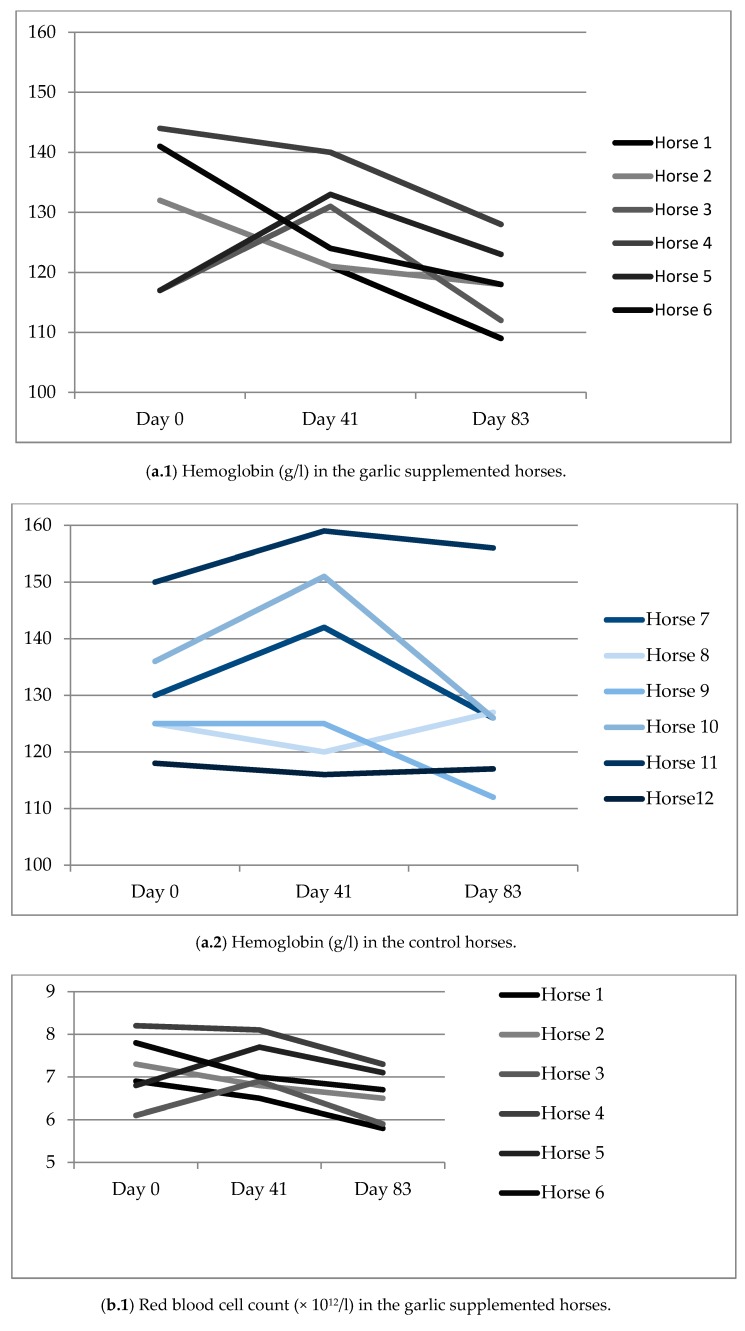

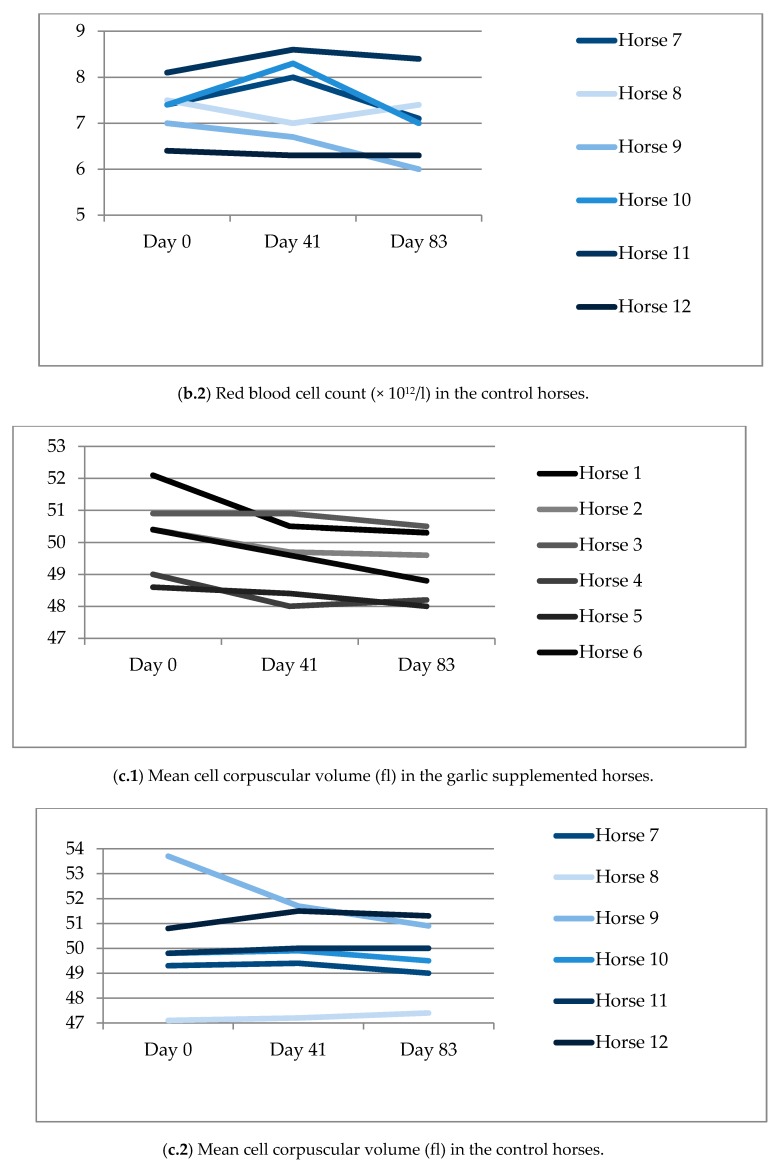
Hematology values of the horses in the garlic supplemented (**a.1**–**c.1**) and control (**a.2**–**c.2**) horses.

There were no statistically significant differences in the blood parameters between the groups (Table 4). However, the garlic supplemented horses showed a slight declining trend in Hb, HcT and RBC values during the study course, but the control horses remain higher values. The means of days 41 and 83 as well as the final Hb, HcT and RBC values on the day 83 in the supplemented horses were numerically smaller compared to the control horses, and the mean final Hb value of the garlic supplemented horses was at the lowest limit of the normal range for Finnhorses [27,28], or even below it. Regarding the results of individual horses (Figure 1), the Hb and RBC values on both days 41 and 83 were lower than the initial value in four of the six garlic supplemented horses, and five of them showed lower values also on day 83 compared with the initial value. These findings may indicate slight anemia in the garlic supplemented horses.

The other parameters were within the ranges reported for healthy Finnhorses [27,28,29], but the WBC value declined numerically in the garlic supplemented horses. It is not possible to draw any conclusion from this, but WBCs increase in the case of inflammatory diseases [30]. Thus, this result supports the speculation on the beneficial respiratory health impacts of garlic reported above. No differences in the differentiated white blood cells existed (data not shown).

Studies on the effects of garlic supplementation on blood chemistry in controlled environments are scarce. The supplemented amount of dried garlic in the present study (32 mg/kg BW) is within recommended limits, however it is clearly close to the lower limit (15 and 200 mg/kg BW of dried garlic) given by The National Academy of Sciences [21]. 

Pearson et al. [12] showed Heinz body anemia due to the garlic supplementation. Because Heinz bodies and bilirubin were not analyzed in our study, it is not possible to make any conclusions regarding the type of anemia. The above-mentioned authors [12] found that recovery from anemia was largely complete five weeks after the removal of garlic supplementation. The supplementation period in their study was 71 days, but they used only four horses (two supplemented with garlic). They concluded that the toxic effect in their study was caused, at least, by oxidative damage of RBCs (RBC was numerically lower for the garlic supplemented horses in the present study as well). Garlic extracts have also been reported to cause Heinz body anemia in dogs [31] and oxidation of RBC in sheep [32]. In horses urticaria associated with dry garlic feeding has also been reported [33]. Furthermore, the feeding of other *Allium* species (e.g., *A. cepa*) has been reported to result in decreased Hb and HcT levels in some other animal species (pigs, dogs, goats) [34,35,36]. Pierce et al. [37] reported anemia caused by wild onion poisoning in horses.

The decrease in Hb and other hematology values is more critical to oxidatively stressed hard-working horses than horses in light work [30]. The safe limit of garlic supplementation potentially depends on factors such as differences between individual horses, and the health status and exercise level of the horse. Low intake levels (15 mg/kg BW dried garlic) are unlikely to result in a risk of adverse effects in healthy, non-exercising, non-oxidatively stressed adult horses [21]. However, Bergero and Valle [20] pointed out that the form of garlic supplementation (dry, fresh, garlic oil, extract) may contain different substances with different biological effects.

## 4. Conclusions

To our knowledge, this is the first organized study in controlled conditions to show possible positive and negative health effects of garlic supplementation in horses. Although this study shows that dried garlic may help to remove tracheal mucus, it also points out that there may be a risk of adverse effects on hemoglobin levels and red blood cell amount if fed with garlic for long periods of time. The supplementation level (32 mg/kg BW) of dried garlic fed to horses seemed to reduce the tracheal symptoms and accumulation of tracheal exudates, but may also cause decreased hematologic values when fed continuously for a nearly three-month period. However, the small data set and subjective evaluation applied in the present study mean that the results need to be considered as preliminary results. Consequently, further research is needed to identify safe garlic doses and supplementation duration for horses, as well as to examine the positive and preventive health effects.

## Figures and Tables

**Table 1:** Monthly averages of the stable air temperature and humidity.

Housing Conditions	November	December	January
Temperature °C			
Mean	9.6	7.3	10.7
Range	8–11	3–10	4–12
Humidity %			
Mean	71.5	65.1	66.8
Range	58–82	46–82	43–85

**Table 2:** Numbers of horses with endoscopy examination findings in the two groups.

Experimental Group; Horse	Initial (Day 0)	Day 41	Day 83	Control Group; Horse	Initial (Day 0)	Day 41	Day 83
1	1	0	1	7	1	1	1
2	1	0	0	8	0	1	0
3	1	1	0	9	1	0	0
4	0	1	0	10	1	1	1
5	1	1	1	11	1	0	1
6	1	0	0	12	0	0	0
Total	5	3	2	Total	4	3	3

1 = findings; 0 = no findings.

**Table 3:** Incidence of neutrophil cells in tracheal mucus in individual horses.

Experimental Group; Horse	Initial (Day 0)	Day 41	Day 83	Control Group; Horse	Initial (Day 0)	Day 41	Day 83
1	++	++	+++	7	+	+++	++
2	–	(+)	–	8	–	–	++
3	+++	++	+	9	++	–	(+)
4	(+)	–	–	10	+++	+++	+++
5	+	+++	+	11	+	+	+++
6	–	–	(+)	12	–	(+)	–

– = none or some single cells; + = single cells and few small pools of cells; ++ = several large pools of cells; +++ = abundant pools of cells.

**Table 4:** Hematology (mean, (s.d)) of the groups during the study period.

Hematology Parameter	Experimental Group			Control Group		
	Initial (Day 0)	Day 41	Day 83	Initial (Day 0)	Day 41	Day 83
Hb, g/L	130.5 (11.5)	128.3 (7.6)	118.0 (7.0)	130.7 (11.2)	135.5 (17.7)	127.3 (15.3)
HcT, %	36.9 (0.0)	35.4 (0.0)	32.3 (0.0)	36.4 (0.0)	37.2 (0.0)	34.9 (0.0)
RBC, × 10^12^/L	7.17 (0.7)	7.15 (0.6)	6.57 (0.6)	7.29 (0.6)	7.46 (1.0)	7.04 (0.8)
MCV, fl	50.0 (1.3)	49.5 (1.1)	49.2 (1.1)	50.1 (2.2)	50.0 (1.6)	49.7 (1.4)
WBC, × 10^9^/L	8.15 (1.3)	7.52 (0.7)	6.97 (0.7)	7.76 (1.3)	6.95 (0.9)	7.52 (1.3)

Hb = hemoglobin; HcT = hematocrit; RBC = red blood cells; MCV = mean cell corpuscular volume; WBC = white blood cells.

## References

[1] Rahman M.S. (2007). Allicin and other functional active components in garlic: Health benefits and bioavailability. Int. J. Food Prop..

[2] McGorum B.C., Ellison J., Cullen R.T. (1998). Total and respirable airborne dust endotoxin concentrations in three equine management systems. Equine Vet. J..

[3] Berndt A., Derksen F.J., Robinson N.E. (2010). Endotoxin concentrations within the breathing zone of horses are higher in stables than on pasture. Vet. J..

[4] Raymond S.L., Curtis E.F., Winfield L.M., Clarke A.F. (1997). A comparison of respirable particles associated with various forage products for horses. Equine Pract..

[5] Vandeput S., Istasse L., Nicks B., Lekeux P. (1997). Airborne dust and aeroallergen concentrations in different sources of feed and bedding for horses. Vet. Q..

[6] Elfman L., Wålinder R., Riihimäki M., Pringle J., Mazzeo D. (2011). Air quality in horse stables. Chemistry, Emission Control, Radioactive Pollution and Indoor Air Quality.

[7] Munday R., Munday C.M. (2001). Relative activities of organosulfur compounds derived from onions and garlic in increasing tissue activities of quinone reductase and glutathione transferase in rat tissues. Nutr. Cancer.

[8] Wade L.L., Newman S.J. (2004). *Hemoglobinuric nephrosis* and *Hepastoplenic eryrhophagocytosis* in a dusky-headed conure (*Aratinga weddeli*) after ingestion of garlic (*Allium sativum*). J. Avian Med. Surg..

[9] Ruwende C., Hill A. (1998). Glucose-6-phosphate dehydrogenase deficiency and malaria. J. Mol. Med..

[10] Harvey J.W., Racker D. (1985). Experimental onion-induced hemolytic anemia in dogs. Vet. Pathol..

[11] Hutchison T.W.S. (1977). Onions as a cause of Heinz body anaemia and death in cattle. Can. Vet. J..

[12] Pearson W., Boermans H.J., Bettler W.J., McBride B.W., Lindinger M.I. (2005). Association of maximum voluntary dietary intake of freeze-dried garlic with Heinz body anemia in horses. Am. J. Vet. Res..

[13] Williams C.A., Lamprecht E.D. (2008). Some commonly fed herbs and other functional foods in equine nutrition: A review. Vet. J..

[14] Borrelli F., Caspasso R., Izzo A.A. (2007). Garlic (*Allium sativum* L.): Adverse effects and drug interactions in humans. Mol. Nutr. Food Res..

[15] Elghandour M.M.Y., Reddy P.R., Salem A.Z.M., Reddy P.P.R., Hyder I., Barbabosa-Pliego A., Yasawini D. (2018). Plant bioactives and extracts as feed additives in Horse nutrition. J. Equine Vet. Sci..

[16] Pearson W. Ethnoveterinary medicine: The Science of botanicals in equine health and disease. Proceedings of the Second Annual European Equine Health and Nutrition Congress.

[17] Kook S., Gun-Hee K., Choi K. (2009). The antidiabetic effect of onion and garlic in experimental diabetic rats: Meta-analysis. J. Medic. Food.

[18] Soffar S.A., Mokhtar G.M. (1991). Evaluation of the antiparasitic effect of aqueous garlic (*Allium sativum*) extract in *Hymenolepiasis nana* and *Giardia*. J. Egypt. Soc. Parasitol..

[19] Cellini L., Di Campli E., Masuli M. (1996). Inhibition of *Helicobacter pyroli* by garlic extract (*Allium sativum*) FEMS. Immunol. Med. Microbiol..

[20] Bergero D., Valle E. (2006). A critical analysis on the use of herbs and herbal extracts in feeding sport horses. Pferdeheilkunde.

[21] The National Academies Report in Brief (2008). Safety of Dietary Supplements for Horses, Dogs and Cats.

[22] Saastamoinen M., Särkijärvi S., Hyyppä S. (2015). Reducing respiratory health risks to horses and workers: A comparison of two stall bedding materials. Animals.

[23] Luke (2018). Feed Tables and Nutrition Recommendations. Natural Resources Institute Finland. https://portal.mtt.fi/portal/page/portal/Rehutaulukot/feed_tables_englishorhttp://urn.fi/URN:ISBN:978-952-326-054-2.

[24] Saastamoinen M., Hellämäki M., Saastamoinen M., Fradinho M.J., Santos A.S., Miraglia N. (2012). Forage analysis as a basis of feeding of horses. Forages and Grazing in Horse Nutrition.

[25] Clarke E.G.C., Clarke M.L. (1967). Garner’s Veterinary Toxicology.

[26] Riihimäki M., Raine A., Elfman L., Pringle J. (2008). Markers of respiratory inflammation in horses in relation to seasonal changes in air quality in a conventional racing stable. Can. J. Vet. Res..

[27] Pösö A.R., Soveri T., Oksanen H.E. (1983). The effect of exercise on blood parameters in Standardbred and Finnish-bred horses. Acta Vet. Scand..

[28] Movet Laboratory Handbook. www.movet.fi.

[29] Saastamoinen M.T. (1994). Propionic acid treated grain (oats) in the diet of horses. Agric. Sci. Finl.

[30] Lindner A. (1998). Laboratory Diagnosis for Sport Horses.

[31] Hu Q., Yang Q., Yamoto O., Yamasiki M., Meade Y., Yoshihara T. (2002). Isolation and identification of organosulfur compounds oxidizing canine erythrocytes from garlic (*Allium sativum*). J. Agric. Food. Chem..

[32] Stevens H. (1984). Suspected wild garlic poisoning in sheep. Vet. Rec..

[33] Miyazawa K., Ito M., Ohsaki K. (1991). An equine case of urticaria associated with dry garlic feeding. J. Vet. Med. Sci..

[34] Ostrowska E., Nicholas K.G., Sterling S.J., Brendan G.T., Rodney B.J., Earling D.R., Jois M., Dunshea F.R. (2004). Consumption of brown onions (*Allium cepa* var. *cavalier* and var. *density*) moderately modulates blood lipids, haematological and haemostatic variables in healthy pigs. Br. J. Nutr..

[35] Ogawa E., Shinoki T., Akahori F., Masaka T. (1986). Effect of onion ingestion on anti-oxidizing agents in dog erythrocytes. Jpn. J. Vet. Sci..

[36] Heidarpour M., Fakrieh M., Aslani M.R., Mohri M., Keywanloo M. (2011). Oxidative effects of long-term onion (*Allium cepa*) feeding on goat erythrocytes. Comp. Clin. Pathol..

[37] Pierce K.R., Joyce J.R., England R.B., Jones P. (1972). Acute hemolytic anemia caused by wild onion poisoning in horses. Am. Vet. Med. Ass. J..

